# The complete chloroplast genome of the *Buddleja alternifolia* (Buddleiaceae), an ornamental plant

**DOI:** 10.1080/23802359.2019.1704186

**Published:** 2020-01-07

**Authors:** Guolun Jia, Weirui Wang, Xiaoqing Liang, Peng Li

**Affiliations:** a Key Laboratory of Resource Biology and Biotechnology in Western China, Ministry of Education, School of Life Sciences, Northwest University, Xi’an, Shaanxi, PR China;; b Department of Pharmacy, Xi’an International University, Xi’an, Shaanxi, PR China

**Keywords:** Chloroplast genome, phylogenetic analysis, Buddleiaceae, *Buddleja alternifolia*

## Abstract

*Buddleja alternifolia* is China’s specialty, and scattered in northwest China. Here, we assembled and characterized the complete chloroplast (cp) genome of *B. alternifolia* using Illumina sequencing data for the first time. The complete cp genome was 154,280 bp in length, consisting of a pair of inverted repeats of 25,440 bp, a large single-copy (LSC) region of 85,330 bp, and a small single-copy (SSC) region of 18,070 bp. The genome encoded 115 unique genes, including 80 protein-coding genes, 31 tRNA genes, and four rRNA genes. Phylogenetic analysis based on 16 complete cp genome sequences indicated that *B. alternifolia* is closely related to *Buddleja colvilei*.

*Buddleja alternifolia* Maxim. is a woody perennial shrub which grows up to 4 m in height and sprouts from May to July, belongs to the Buddleja genus, Buddleiaceae family. It is China’s specialty, scattered in northwest of China, and has been widely used for ornamental purpose. So far, genetic information of *B. alternifolia* was barely reported except for tissue culture (Liu et al. 2009), seed germination and the establishment of seedlings (Han et al. 2013) and drought resistance physiology (Yan et al. 2017). In this study, we assembled and annotated the complete chloroplast (cp) genome sequence of *B. alternifolia* using Illumina sequencing data for the first time.

Fresh leaves of *B. alternifolia* were collected in the Yinchuan Botanical Garden (38°28′N, 106°16′E; Ningxia, NW China). A voucher specimen (2019BA1) is deposited at the key laboratory of resource biology and biotechnology in Northwest University. We used the modified CTAB method to extract the total genomic DNA (Doyle and Doyle 1987). A shotgun library constructed following the manufacturer’s protocol for the Illumina HiSeq X Ten Sequencing System (Illumina, San Diego, CA). We assembled the cp genome using the program MITObim version 1.8 (DSM Nutritional Products Ltd, Kaiseraugst, Switzerland) (Hahn et al. 2013), with that of *Buddleja colvilei* (GenBank: *MH411147*) as the initial reference. To validate the assembly, polymerase chain reaction (PCR) amplifications and Sanger sequencing were conducted to verify the four junctions between inverted repeats (IRs) and large single-copy region (LSC)/small single-copy region (SSC). The cp genome annotation was performed using DOGMA (Wyman et al. 2004), coupled with manual correction for protein-coding region (CDS) boundaries. The web-based tool OGDRaw version 1.2 (http://ogdraw.mpimp-golm.mpg.de/) was employed to generate a map of the complete cp genome (Lohse et al. 2013).

The complete cp genome sequence of *B. alternifolia* has been submitted to GenBank (accession number *MN623351*). The complete cp genome was 154,280 bp in length, consisting of a pair of inverted repeat regions of 25,440 bp each, an LSC region of 85,330 bp, and a small single SSC of 18,070 bp. A total of 115 genes were annotated, including 31 tRNA, 4 rRNA, and 80 protein-coding genes. The overall GC content of the cp genome was 38.0%. In addition, 10 PCG genes (*atpF*, *ndhA*, *ndhB*, *petB*, *petD*, *rpl2*, *rpl16*, *rpoC1*, *rps12,* and *rps16*) possess a single intron, 68 PCG genes no intron, 2 other genes (*clpP* and *ycf3*) harbor two introns. The 6 tRNA genes (*trnA-UGC*, *trnG-CGA*, *trnI-GAU*, *trnK-UUU*, *trnL-UAA,* and *trnV-UAC*) harbor a single intron.

To reveal the phylogenetic position of *B. alternifolia* within the Buddleiaceae, the neighbor-joining method was used by MEGA version 7.0 with 1000 bootstrap replicates (Kumar et al. 2016) (http://www.megasoftware.net/). Depending on the available data from GenBank, we selected 16 other complete cp genomes of species from the Buddleiaceae. Our result confirmed that the *B. alternifolia* closely related to *B. colvilei* (Figure 1).

**Figure 1.**
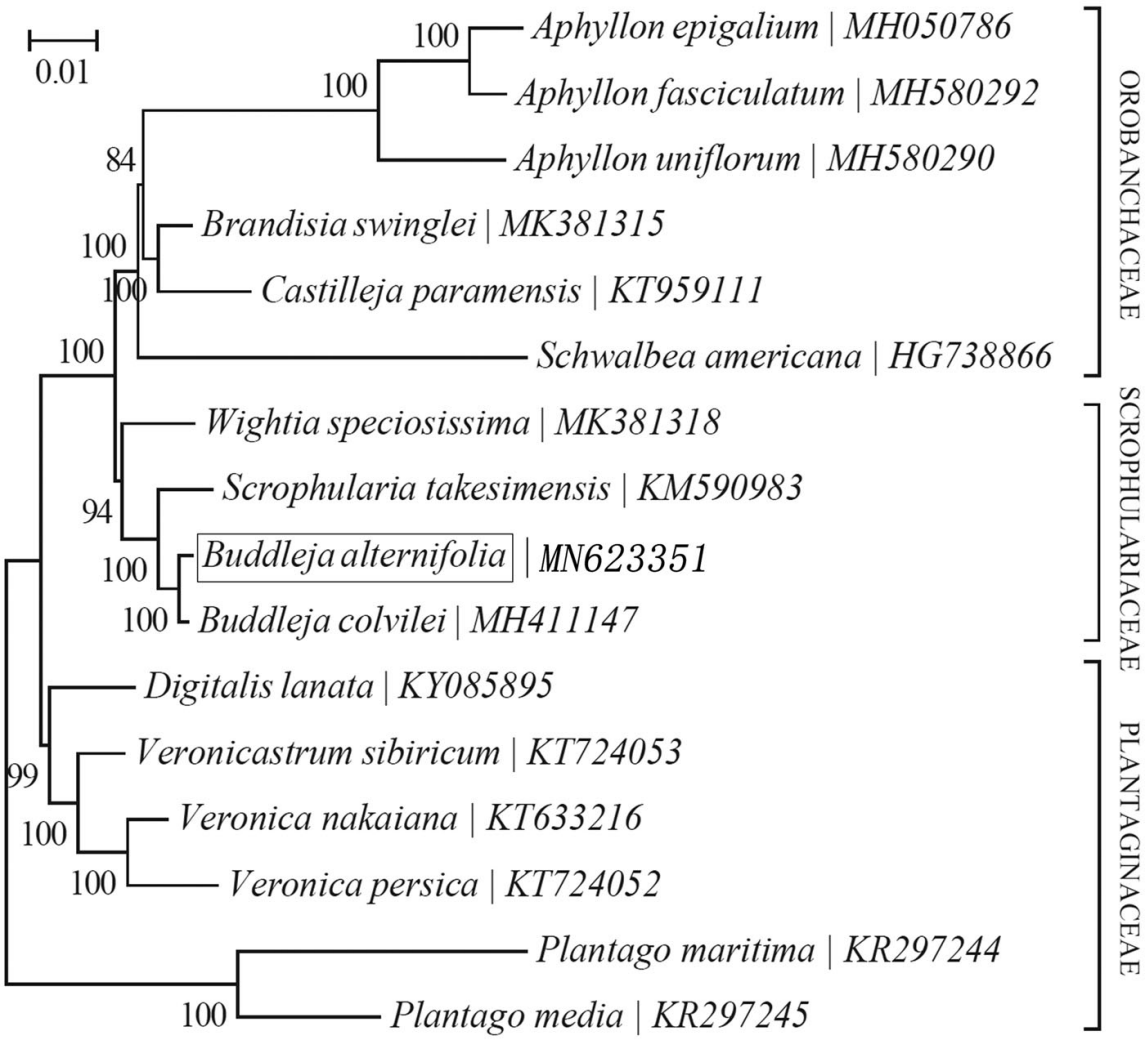
Phylogenetic relationship of the *Buddleja alternifolia* chloroplast genome with 15 previously reported complete chloroplast genomes. The numbers at the tree nodes indicate the percentage of bootstrapping after 1000 replicates.

The complete cp genome of *L. maackii* can be used for further phylogenomic studies of Buddleiaceae and will provide fundamental information to effectively conserve important species.
